# The modulation of TRPM7 currents by nafamostat mesilate depends directly upon extracellular concentrations of divalent cations

**DOI:** 10.1186/1756-6606-3-38

**Published:** 2010-12-01

**Authors:** Xuanmao Chen, Tomohiro Numata, Minghua Li, Yasuo Mori, Beverley A Orser, Michael F Jackson, Zhi-Gang Xiong, John F MacDonald

**Affiliations:** 1 Department of Physiology, University of Toronto, Canada; 2 Robarts Research Institute, University of Western Ontario, Canada; 3 Robert S. Dow Neurobiology Laboratories, Legacy Research, Portland, USA; 4 Sunnybrook Health Sciences Centre, University of Toronto, Canada; 5 Department of Synthetic Chemistry and Biological Chemistry, Graduate School of Engineering, Kyoto University; 6 CREST, JST, Kyoto 615-8510, Japan

## Abstract

Concentrations of extracellular divalent cations (Ca^2+ ^and Mg^2+^) fall substantially during intensive synaptic transmission as well as during some pathophysiological conditions such as epilepsy and brain ischemia. Here we report that a synthetic serine protease inhibitor, nafamostat mesylate (NM), and several of its analogues, block recombinant TRPM7 currents expressed in HEK293T cells in inverse relationship to the concentration of extracellular divalent cations. Lowering extracellular Ca^2+ ^and Mg^2+ ^also evokes a divalent-sensitive non-selective cation current that is mediated by TRPM7 expression in hippocampal neurons. In cultured hippocampal neurons, NM blocked these TRPM7-mediated currents with an apparent affinity of 27 μM, as well as the paradoxical Ca^2+ ^influx associated with lowering extracellular Ca^2+^. Unexpectedly, pre-exposure to NM strongly potentiated TRPM7 currents. In the presence of physiological concentrations of extracellular divalent cations, NM activates TRPM7. The stimulating effects of NM on TRPM7 currents are also inversely related to extracellular Ca^2+ ^and Mg^2+^. DAPI and HSB but not netropsin, blocked and stimulated TRPM7. In contrast, mono-cationic, the metabolites of NM, p-GBA and AN, as well as protease inhibitor leupeptin and gabexate failed to substantially modulate TRPM7. NM thus provides a molecular template for the design of putative modulators of TRPM7.

## Background

The eight members of the transient receptor potential melastatin (TRPM) group represent a subclass of non-selective cation transient receptor potential (TRP) channels [1,2]. One of these, the TRPM7 channel, is widely expressed in various locations throughout the body and, it is also expressed within the central nervous system. The function of TRPM7 channels is usually attributed to one of Mg^2+ ^homeostasis [3,4] but they are also a source of entry of Ca^2+ ^and contribute to the death of hippocampal neurons following ischemia [5-7]. These channels are also mechano-sensitive [8], and they contribute to mechanisms controlling vesicular release of acetylcholine from sympathetic neurons [9].

Although TRPM7 channels are permeable to both Ca^2+ ^and to Mg^2+^, the extracellular presence of these divalent cations strongly inhibits the influx of monovalent cations, resulting in strong outward rectification of TRPM7-mediated currents [3,4]. As a consequence, in the presence of physiological concentrations of these divalent cations, inward currents are almost negligible. Oxidative stress induced by periods of oxygen/glucose deprivation enhances TRPM7 currents in cultured hippocampal and cortical neurons and entry of Ca^2+ ^via these channels can lead to delayed death of neurons [5,7]. Both the RNA message for TRPM7 and the protein itself are expressed in cultured hippocampal neurons as well as in CA1 pyramidal neurons of the rat [5,7]. Nevertheless, it can be difficult to detect inward TRPM7-mediated currents in single neurons because their contribution can be small relative to the large variety of voltage-dependent currents found in these cells and identification of TRPM7 current is further impeded by the lack of selective channel blockers [6]. An alternative approach is to accentuate monovalent cation influx through TRPM7 channels simply by lowering extracellular concentrations of divalent cations [6,10]. This is not just a procedural maneuver, as large decreases in extracellular concentrations of Mg^2+ ^and Ca^2+ ^[11,12] are characteristic of brain ischemia. In acutely isolated and *in situ *CA1 neurons TRPM7 currents can be characterized using this divalent lowering protocol together with RNAi techniques. We were able to use this approach to show that reducing TRPM7 currents in CA1 neurons of the rat provides substantial protection against the loss of CA1 neurons in a model of global ischemia [5,7].

Protons can compete with Ca^2+ ^and Mg^2+ ^for binding to the channel pore region and thereby relieve the channel block of monovalent cations [13,14]. Decreasing pH from 7.4 to 4.0 reduces the IC_50 _values for monovalent cation block by 510 and 410 fold for Mg^2+ ^and Ca^2+^, respectively [15]. The sensitivity of monovalent conductance to block by Ca^2+ ^and Mg^2+ ^in human TRPM7 channels is directly dependent upon the negative charge provided by two residues, Glu-1052 and Asp-1054, suggesting that divalent cations bind to site incorporating these two residues [15]. To date, there are few selective pharmacological tools to study the physiological functions of TRPM7. As TRPM7 currents are blocked by extracellular Ca^2+ ^and Mg^2+^, we hypothesized that dicationic chemicals could mimic the effects of divalent cations in blocking TRPM7 channels. Here we report that the dications NM, DAPI and HSB function as novel regulators of TRPM7 currents in hippocampal neurons, likely by competing for divalent cations and thereby controlling the entry of Ca^2+ ^and Mg^2+^. In the absence of extracellular divalent cations, NM caused a potent and voltage-dependent inhibition of TRPM7 currents which mimicked the effects of the divalent cations themselves. Moreover, the NM-induced inhibition was occluded as the extracellular concentrations of divalents were increased suggesting that NM competes with divalent cations for the block of TRPM7 channels.

## Methods

### TRPM7 Expressed in HEK293T Cells

Electrophysiological recordings were made from HEK293T cells transfected wild-type (WT) human TRPM7 or the E1052A, and the D1054A mutant channels, as previously described [8,13].

Whole-cell and patch recordings were performed on HEK293T cells at room temperature (22-25°C) with Axopatch 200B (Molecular Devices, Sunnyvale, CA) amplifier. For whole-cell recordings, the Cs^+^-based bath solution contained (in mM) 100 Cs-aspartate, 1 EGTA, 10 N-2-Hydroxyethylpiperazine-N'-thanesulfonic acid (HEPES) and 120 mannitol (pH adjusted to 7.4 with CsOH). When required, 1 mM ethyleneglycol-bis-(α-amino-ethyl ether) N,N'-tetra-acetic acid (EGTA) was replaced with added CaCl_2 _and MgCl_2_ as indictated in results section. The pipette solution contained 100 Cs-aspartate, 1 EGTA, 10 HEPES, 0.5 CsCl, and 100 mannitol (pH 7.4). The inhibition ratio (%) was calculated according to the following equation: inhibition ratio (%) = 100 × [1 - (I_NM_)/(I_Ctl_)], where I_Ctl _is whole-cell current observed before application of NM at +100 and -100 mV. I_NM _represents the current observed during application of NM.

### Primary cultures of mouse hippocampal and cortical neurons

Time-pregnant Swiss mice (embryonic day 16) were anesthetized with halothane and were decapitated. Brains of fetuses were removed rapidly and placed in Ca^2+ ^and Mg^2+ ^free ice cold PBS. Whole hippocampus or cerebral cortices were dissected under a dissection microscope and incubated with 0.05% trypsin-EDTA for 10 min at 37°C, then triturated with fire-polished glass pipettes. Cells were counted and plated in poly-L-ornithine-coated culture dishes at a density of 1 × 10^6 ^cells per 35 mm diameter dish. Neurons were cultured with neurobasal medium supplemented with B27 and maintained at 37°C in a humidified 5% CO_2 _atmosphere incubator. Cultures were fed twice a week. Neurons at 14-21 day culture stages were used for electrophysiological recordings and calcium image.

### Electrophysiological recording on cultured hippocampal or cortical neurons

Electrophysiological recordings were made from cultured mouse hippocampal or cortical neurons, 14-20 days after plating, which were grown as described in [16]. The extracellular solution (ECF) was composed of (mM) 140 NaCl, 2 CaCl_2_, 1 MgCl_2_, 25 HEPES, 33 glucose, 5.4 KCl and 0.0002 tetrodotoxin with pH of 7.3-7.4 and osmolality ranging from 320-330 mOsm. The intracellular solution for voltage clamp recording consisted of (mM) 140 CsF, 11 EGTA as intracellular Ca^2+ ^chelating buffer, 10 HEPES, 2 MgCl_2_, 2 tetraethyl ammonium chloride (TEA-Cl), 1 CaCl_2_, and 4 K_2_ATP. Pipette resistance ranges were 2-4 MΩ when filled with this intracellular solution. All recordings were performed at room temperature. Membrane potential was held at -60 mV throughout the recording if not otherwise indicated. Access resistance was monitored by applying a voltage step of -5 mV. Low divalent cation-induced currents were elicited by rapid application of low Ca^2+ ^solution (0.1 mM Ca^2+ ^and 0.1 mM Mg^2+ ^if not otherwise stated) delivered from a multi-barrelled fast perfusion system for 5 seconds and repeated every minute. The perfusion rate of the solution was approximately 1 ml per minute. Whole-cell currents were recorded using an Axopatch-1D amplifier (Molecular Devices, Sunnyvale, CA). Cell-attached loose-patch recordings were also preformed as described previously [6,17]. In those recordings, pipettes had resistances of 0.8-2 MΩ and were filled with control extracellular solution. Electrophysiological recordings were filtered at 2 kHz and digitized at 5-10 kHz using a Digidata 1332A (Molecular Devices, Sunnyvale, CA) or/and simultaneously through MiniDigi 1A (Molecular Devices, Sunnyvale, CA), and acquired online with pClamp8.2 (Molecular Devices, Sunnyvale, CA) or/and Axoscope9.2 (Molecular Devices, Sunnyvale, CA).

### Calcium imaging

Fura-2 fluorescent Ca^2+ ^imaging was performed as described previously [18]. Cortical or hippocampal neurons grown on 25 mm round glass coverslips were washed three times with ECF and incubated with 5 μM Fura-2-AM for ~ 40 min at room temperature. Neurons were then washed three times and incubated in normal ECF for 30 min. Coverslips with Fura-2-loaded neurons were transferred to a perfusion chamber on the stage of an inverted microscope (Nikon TE300, Tokyo, Japan). Cells were illuminated using a xenon lamp (75W) and observed with a 40 × UV fluor oil-immersion objective lens. Video images were obtained using a cooled CCD camera (Sensys KAF 1401, Photometrics, Tucson, AZ) mounted on an inverted microscope (Nikon TE300, Tokyo, Japan). Digitized images were acquired, stored, and analyzed in a PC controlled by Axon Imaging Workbench software (AIW2.1, Molecular Devices, Sunnyvale, CA). The shutter and filter wheel (Lambda 10-2, Sutter Instrument, Novato, CA) were also controlled by AIW to allow timed illumination of cells at 340 and 380 nm excitation wavelengths. Fura-2 fluorescence was detected at an emission wavelength of 510 nm. Ratio images of 340/380 nm were analyzed by averaging pixel ratio values in circumscribed regions of cells in the field of view. The values were exported from AIW to SigmaPlot (Jandel Scientific, Ekrath, Germany) for further analysis and plotting.

### Data analysis

Data were analyzed with Clampfit 9.2. (Molecular Devices, Sunnyvale, CA), Excel 2002 (Microsoft Corporation, Redmond, WA), Origin 5.0 (OriginLab Corp., Northhampton, MA) and finalized illustrated using CorelDraw X3 (Corel Corporation; Ontario, Canada). Currents were normalized to the amplitude of control responses. NM or DAPI inhibitory concentration-response plots were fitted to the logistic equation: *I *= (A_max_-A_0_)/[1+(X/IC_50_)^n^]+A_0_, where *I *is the normalized current amplitude, X is the antagonist concentration; n is Hill coefficient; IC_50 _is the concentration of antagonist that generate 50% of maximal inhibition. Results are reported for the text, the figures and amplitude histograms, as mean ± S.E.M. They represent the mean of *n *individual measurements on different cells. Statistical analysis was done with the unpaired or paired *t*-test, when appropriate. Chemicals are re-drawn based on the structures downloaded from Pubchem. NM was purchased from BioMol (Plymouth Meeting, PA) and its metabolites p-GBA and AN were from TCI and gabexate, DAPI, HSB from Sigma (St. Louis, MO).

## Results

### NM is a blocker of recombinant TRPM7 currents expressed in HEK293T cells

**Figure 1:**
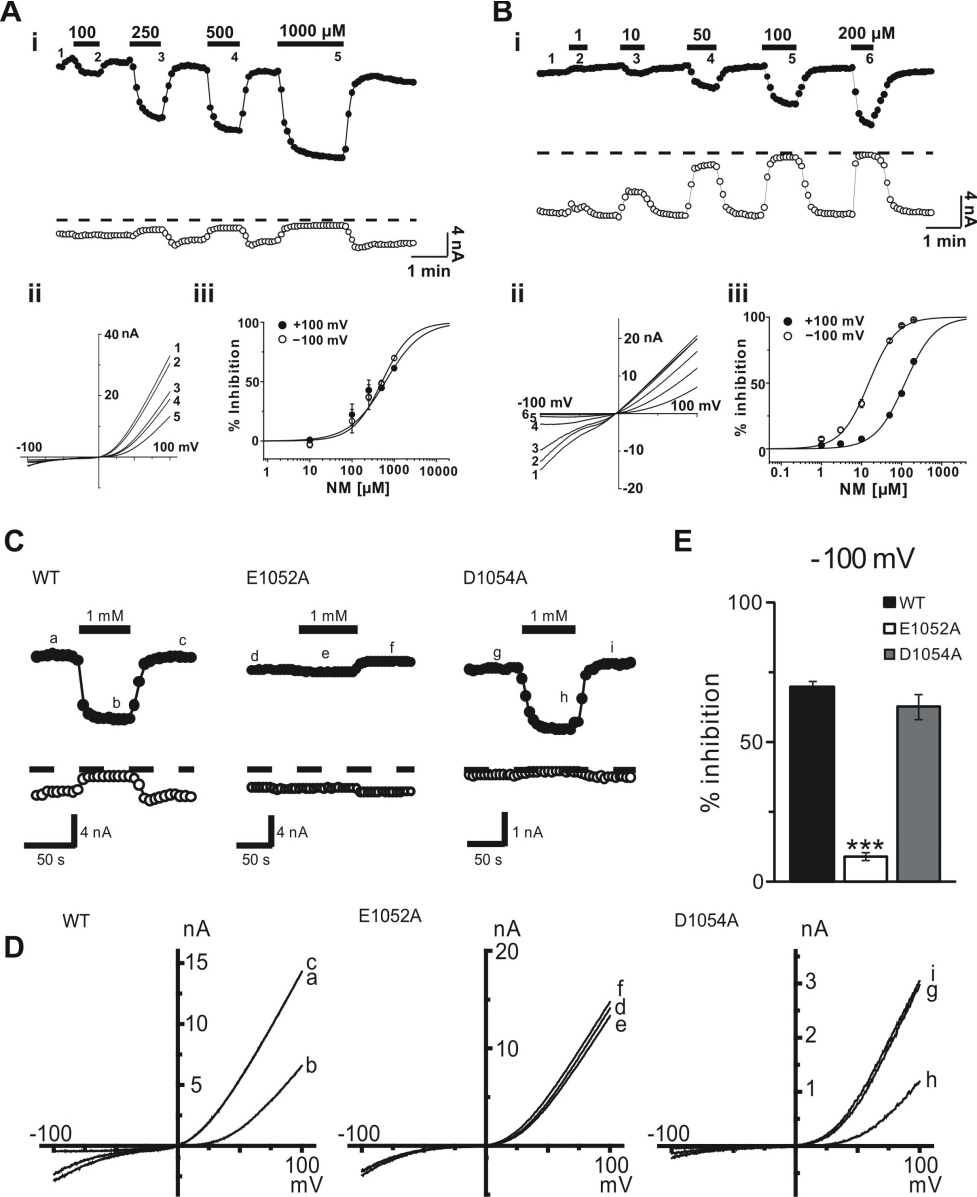
**NM reversibly inhibits TRPM7 currents in HEK293T cells**. A i, example peak currents recorded at +100 (filled circle) and -100 mV (open circle) (V_h _= 0 mV) in the presence of 0.1 mM Ca^2+ ^and 0.1 mM Mg^2+ ^before and during NM: 100 μM, 250 μM, 500 μM, and 1 mM (filled bars). ii, example *I*-*V *curve for NM-induced inhibition of TRPM7 (+100 mV; IC_50_: 617 ± 40 μM; Hill coefficient: 1.4; n = 14 and at -100 mV; IC_50_: 514 ± 39 μM; Hill coefficient: 1.3; n = 14. B. i, example peak currents recorded at +100 (filled circle) or -100 mV (open circle) (V_h _= 0 mV) in divalent free solution before and during NM: 1 μM, 10 μM, 50 μM, 100 μM, and 200 μM (filled bars). ii, example *I*-*V *curves of currents before (at *1 *in *A*) and during applications of NM (at *2, 3, 4, 5 *and *6 *in A). iii, dose-response curve of inhibition by NM at +100 mV; IC_50_: 121 ± 2 μM; Hill coefficient: 1.2; n = 6 and at -100 mV; IC_50_: 15 ± 2 μM; Hill coefficient: 1.3; n = 6. C, example peak currents recorded at +100 (filled circle) and -100 mV (open circle) (V_h _= 0 mV) in the presence of 0.1 mM Ca^2+ ^and 0.1 mM Mg^2+ ^before (a, d, g), during (b, e, h) and after (c, f, i) 1 mM NM. D, example *I-V *curves for WT TRPM7, E1052A, and D1054A mutants. E, inhibition of the average current density of inward currents mediated by WT TRPM7 or its mutants, recorded at -100 mV. Each column represents the mean ± s.e.m. (n = 7-14), ***, P < 0.001 vs. WT.

Voltage ramps were employed to evoke currents from over-expressed TRPM7 channels induced in HEK293T cells. NM failed to inhibit TRPM7 currents in the presence of 1 mM Ca^2+ ^and Mg^2+ ^(not shown). However, when extracellular divalents were reduced to 0.1 mM, NM inhibited outward and inward TRPM7 currents with IC_50 _values of 617 μM at +100 mV and 514 μM at -100 mV (n = 11-14) (Figure 1A). Recordings in divalent-free cation solution further increased the inhibitory potency of NM by a factor of about 5 fold at +100 mV and a factor of 30 fold at -100 mV (Figure 1B) (IC_50 _= 121 μM at +100 mV and IC_50 _= 15 μM at -100 mV). Thus, the inhibition by NM depends strongly upon the concentration of extracellular divalents, and the blockade of inward monovalent cation current. The negative amino acid residues at 1052 and 1054 are important for the inhibition of monovalent cations in TRPM7 channels by divalent cations [15]. In this respect, the inhibition by NM of currents was strongly depressed in E1052A, but not D1054A expressing HEK293T cells (Figure 1C &1D), illustrating that that NM inhibition is dependent upon this site.

### NM blocks TRPM7 currents in cultured Hippocampal Neurons

**Figure 2:**
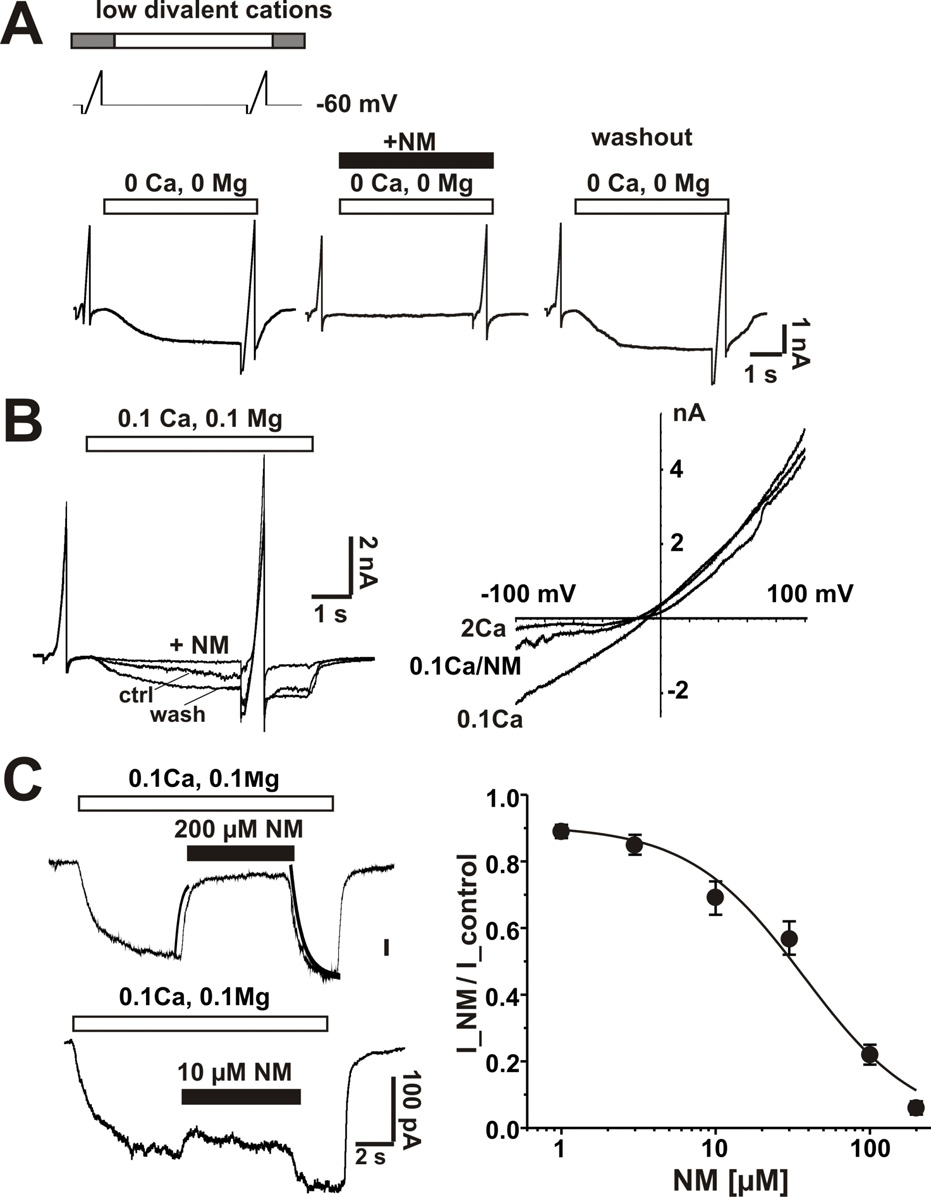
**NM completely and reversibly inhibits TRPM7 current in cultured mouse hippocampal neurons**. A, top, experimental protocol showing voltage ramps (-100 to +100 mV) and applied low divalent cations. TRPM7 was elicited by lowing divalent cations from 2 mM Ca^2+^, 1 mM Mg^2+ ^to indicated lower concentrations. Bottom, current trace examples of 0 Ca^2+ ^and 0 Mg^2+^-induced TRPM7 current at the presence and absence of NM. Co-application of NM (200 μM) reversibly blocked TRPM7 evoked by zero divalent cations. n = 6. B, NM (200 μM) completely blocks TRPM7 evoked by 0.1 mM Ca^2+ ^(and 0.1 mM Mg^2+^). Example current traces superimposed (left) and *I-V *curve (right). n = 8. C, concentration response of NM blockade of TRPM7 current, Left, currents trace examples. NM was acutely applied in the presence of 0.1 mM Ca^2+ ^and 0.1 mM Mg^2+^. The onset time constrant of NM inhibition (200 μM) was 0.29 ± 0.04 s (n = 9) and the offset time constant was 1.07 ± 0.20 s (n = 9). Exponential fitting curves (red) were inserted with the trace (when NM is 200 μM). Right, concentration response curve of NM inhibition; IC_50_: 27 ± 6 μM; hill coefficient: 1.4; n = 6.

In recordings from cultured hippocampal neurons, TRPM7 currents cannot be easily distinguished from a variety of outwardly rectifying currents [6]. However, the rectification of inward TRPM7 currents recorded at hyperpolarized potentials can be dramatically reduced by lowering the concentration of extracellular divalent cations; and, this is indicative of the block of inward cation flux by divalent cations [5,6]. Therefore, we employed applications of extracellular solutions deficient in divalent cations to transiently evoke inward TRPM7 currents, with membrane potential clamped at -60 mV. Extracellular divalents were set at 2 mM Ca^2+ ^and 1 mM Mg^2+^. We first tested if NM blocks responses of cultured hippocampal neurons to applications of solutions lacking any added Ca^2+ ^or Mg^2+^. This solution evoked inward currents that were reversibly blocked by the co-application of NM (200 μM) (Figure 2A). The current-voltage (*I-V*) curve determined before and during application of NM demonstrated that the blockade of these currents was strongly voltage-dependent. Since this zero divalent cation solution has uncertain concentrations of Ca^2+ ^and Mg^2+^, we thereafter used a solution containing added divalent cations (100 μM Ca^2+^, 100 μM Mg^2+^). NM similarly blocked inward currents evoked by this low divalent cation solution. The IC_50 _for the block by NM was 27 μM (Figure 2C). Both the onset and the recovery from the inhibition occurred rapidly suggesting that NM directly blocks the channels underlying the current. Although, this result paralleled what was observed with over expression of TRPM7 channels, it occurred at a much higher concentration of extracellular divalent cations.

### NM inhibits TRPM7-induced increases in intracellular Ca^2+ ^in cultured hippocampal and cortical neurons

**Figure 3:**
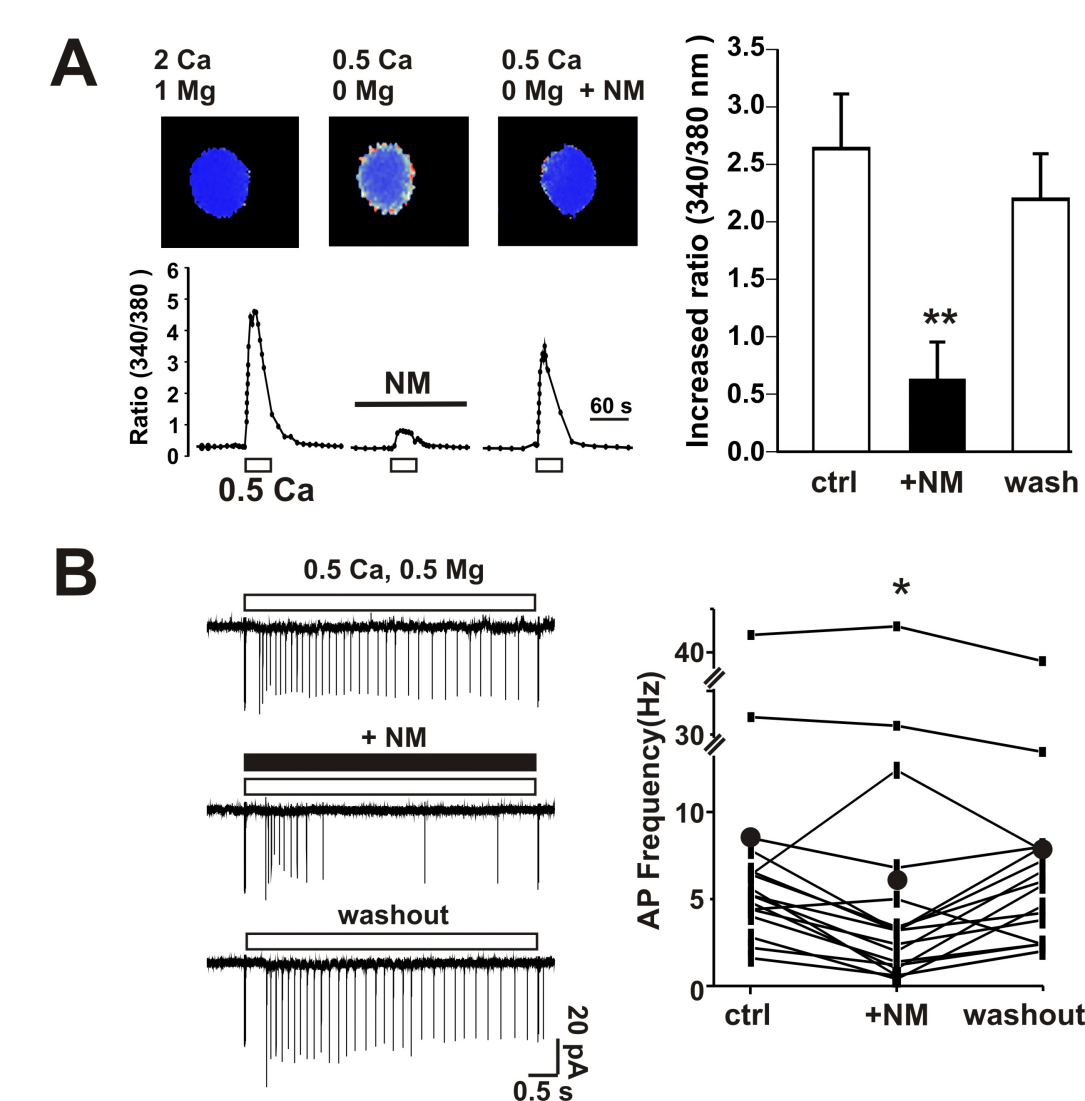
**NM attenuates low-divalent-cation-induced Ca^2+ ^influx and action potentials in cultured hippocampal neurons**. A, calcium image; representative changes in ratio images (top) and 340/380 nm ratio values (bottom) by lowering Ca^2+ ^from 2 mM to 0.5 mM and Mg^2+ ^from 1 mM to 0 mM. NM (50 μM) was co-applied with low divalent cations. B, summarized data shows the reduction of low divalent-cation-induced increase of 340/380 nm ratio by NM, **p < 0.01. n = 11. B. NM (200 μM) reduced the frequency of action potentials in on-cell loose patch induced by lowering divalent cations. Action potentials were induced by 0.5 mM Ca^2+ ^and 0.5 mM Mg^2+^. Left, trace examples; right, statistic analysis. Black circle shows the mean of firing frequency. Firing frequency was significantly (p < 0.05) reduced from 8.5 ± 2.7 Hz (control) to 6.8 ± 2.9 Hz (adding NM) and back to 8.0 ± 2.4 Hz after washing out NM, n = 16.

Reducing extracellular Ca^2+ ^paradoxically induces Ca^2+ ^influx into hippocampal neurons via TRPM7 channels [16,19]. To test if NM inhibits this influx of Ca^2+^, we used Ca^2+ ^imaging techniques to monitor Ca^2+ ^entry. In cultured hippocampal neurons a decrease of extracellular Ca^2+ ^from 2.0 mM to 0.5 mM (absence of added Mg^2+^) induced a net increase of the 340/380 ratio by 2.64 ± 0.48 (n = 11, p < 0.01). Bath perfusion of NM (50 μM) reduced the 340/380 ratio to 0.61 ± 0.32 (n = 9, p < 0.01, Figure 3A). A similar inhibition was observed in cultured mouse cortical neurons where bath perfusion of NM (50 μM) reduced the net increase of 340/380 ratio nm from 1.17 ± 0.03 to 0.20 ± 0.08 (n = 5, p < 0.05, not shown). However, NM (50 μM) did not affect the increase of intracellular Ca^2+ ^induced by applying KCl (50 mM). The net increase of 340/380 ratio was 1.67 in the absence of NM, and it was 1.84 in its presence illustrating that NM does not inhibit the rise in Ca^2+ ^entry simply by preventing depolarization.

### NM attenuates low calcium-induced neuronal excitation

Lowering extracellular Ca^2+ ^potently increases neuronal excitation by eliciting an inward current [10,16,17]. We therefore tested if NM, by blocking TRPM7 currents, could affect neuron excitation upon reducing Ca^2+^. To exclude the involvement of changes in seal conductance due to low extracellular Ca^2+ ^concentration, we used on-cell loose patch technique [6] to record on-cell inward current spikes reflecting action potentials. Since neuronal excitation can be detected with decrease from physiological concentrations of as little as 100 μM [10], we reduced the Ca^2+ ^concentration to 0.5 mM. Figure 3B shows neuron firing was increased upon application of 0.5 Ca^2+ ^and NM attenuated neuron firing frequency from 8.5 ± 2.7 Hz to 6.8 ± 2.9 Hz (p < 0.05, n = 16) illustrating that NM dampens the excitation by blocking the low Ca^2+^-induced current.

### NM also activates TRPM7 currents?

**Figure 4:**
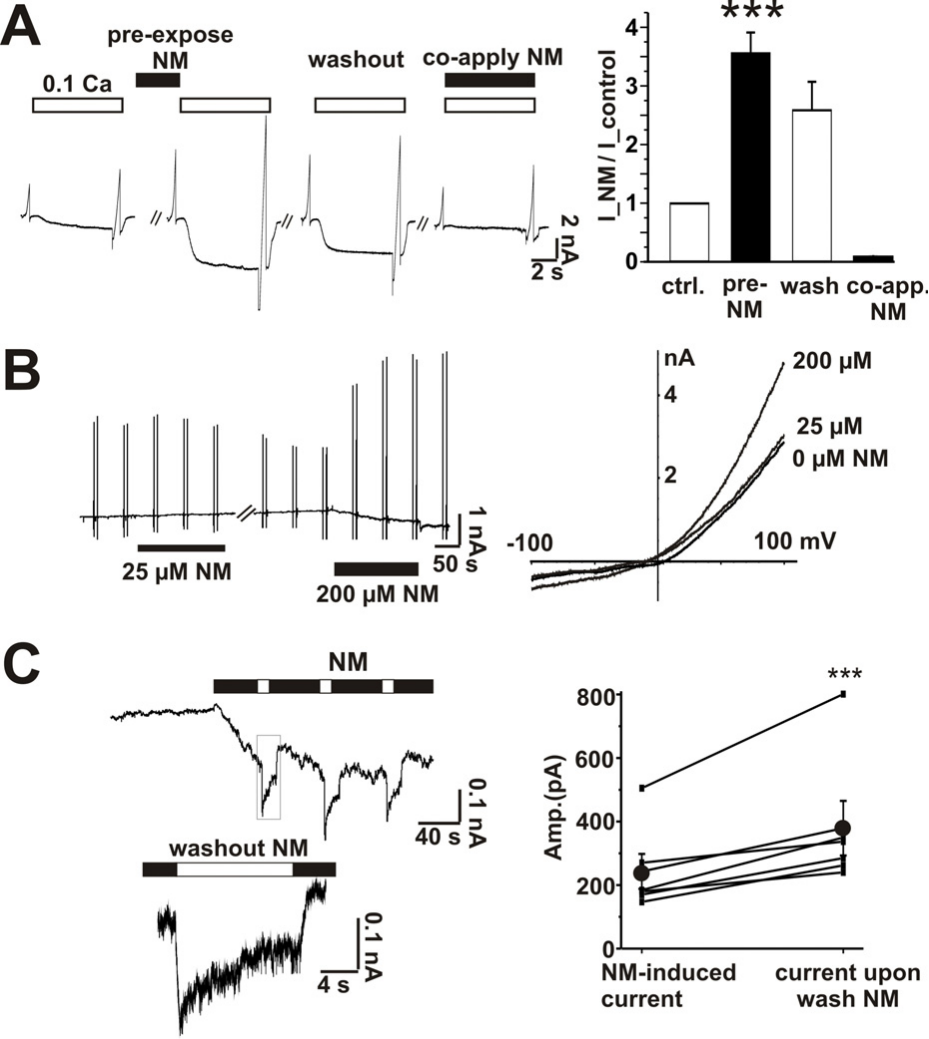
**NM potentiates and also activates TRPM7 channels in hippocampal neurons. A, left, trace examples of the TRPM7 currents**. TRPM7 was evoked by 0.1 mM Ca^2+ ^(and 0.1 mM Mg^2+^). Pre-expose: NM (200 μM) was applied before lowering divalent cations for 1-2 minutes; co-apply: NM (200 μM) was applied together with 0.1 mM Ca^2+ ^(and 0.1 mM Mg^2+^). Right, bar graph showing the potentiated ratio by NM on the TRPM7. n = 7, ***, p < 0.01 vs. control. B, NM opens TRPM7 at the physiological concentration of divalent cations. Left, examples of TRPM7 current trace monitored by voltage ramps (-100 mV to 100 mV). Divalent cation concentration was 2 mM Ca^2+ ^and 1 mM Mg^2+^. Right, *I-V *curve from voltage ramps. 200 μM NM caused marked while 25 μM NM have little effects on TRPM7, n = 6. C, NM activates and also blocks TRPM7 channel. Left, trace example of NM-induced current. Bath solution contained 2 mM Ca^2+ ^and 1 mM Mg^2+ ^and stayed unchanged. Black bar indicates application of NM and white bar indicates washout of NM. NM (200 μM) at the physiological concentration of cations directly induces an inward current, which became larger upon brief NM removal (inset enlarged). n = 5. Right, bar graph showing amplitude of NM-induced current (243 ± 66 pA) compared to current amplitude upon NM washout (379 ± 86 pA).

Next we examined the effect of pre-applying NM on the response to low divalent cations. Unexpectedly, rather than inhibiting the currents pre-applied NM (200 μM) dramatically potentiated evoked currents by about 3.6 ± 0.4 fold (n = 7, p ≤ 0.01) (Figure 4A). This occurred even though simultaneous applications of NM entirely blocked responses to lowered divalent cations (Figure 4A). This finding prompted us to determine whether or not NM might be capable of activating TRPM7 currents in the presence of physiological concentrations of divalent cations. Indeed, applications of NM (200 μM) (n = 6), in the presence of 2 mM Ca^2+ ^and 1 mM Mg^2+^, induced small inward currents (Figure 4B). Voltage ramps demonstrated that this NM-induced current reversed at 0 mV. However, a concentration of NM close to IC_50 _for the block of TRPM7 currents was without effect (Figure 4B).

**Figure 5:**
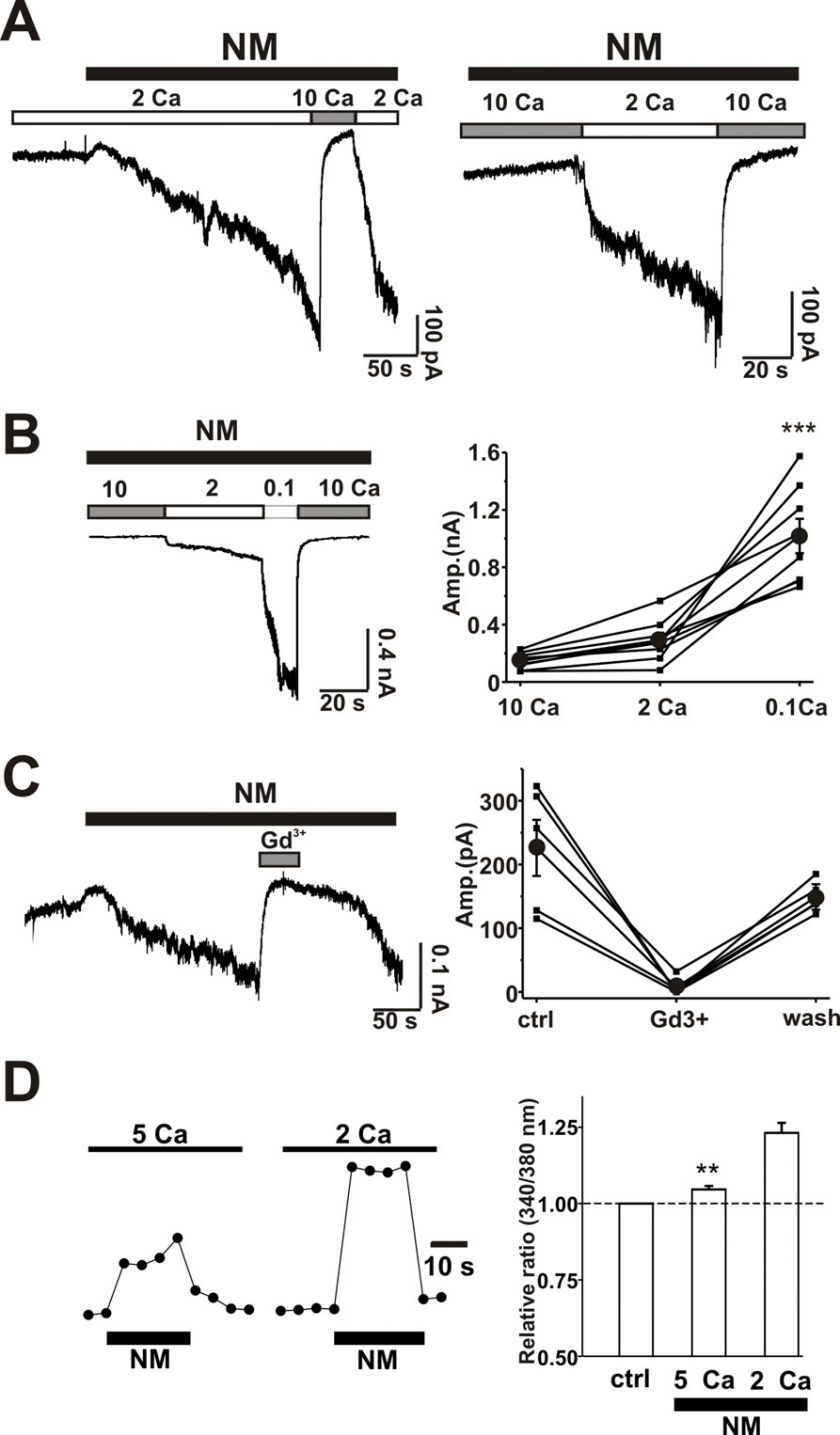
**NM-activated TRPM7 in hippocampal neurons depends on extracellular Ca^2+ ^concentration**. A, right, 10 mM Ca^2+ ^blocks NM (200 μM)-induced TRPM7 current, n = 7. Left, high Ca^2+ ^(10 mM) but not 2 mM Ca^2+ ^occlude the TRPM7 activation by NM (200 μM), n = 6. B, left, NM elicited almost no current at 10 mM Ca^2+ ^(with 1 mM Mg^2+^), small TRPM7 current at 2 mM Ca^2+ ^(with 1 mM Mg^2+^) and large TRPM7 current at 0.1 Ca^2+ ^(with 0.1 Mg^2+^) (n = 8). Right, bar graph shows statistics of NM-induced current at various Ca^2+ ^conditions. The current amplitudes were 147 ± 20 pA at 10 mM Ca^2+^; 291 ± 52 pA at 2 mM Ca^2+ ^and 1018 ± 120 pA at 0.1 mM Ca^2+^. C, TRPM7 current activated by NM was inhibited 97 ± 2% by Gd^3+ ^(10 μM), n = 5. Left, current-trace example; right, bar graph showing statistics of current amplitude. D, NM-induced Ca^2+ ^entry in hippocampal neurons is attenuated by high extracellular Ca^2+^. NM (200 μM)-induced Ca^2+ ^entry monitored by Ca^2+ ^imaging and high extracellular Ca^2+ ^(5 mM) attenuated the Ca^2+ ^entry. **, p < 0.01, 2 mM versus 5 mM Ca^2+^, n = 22. Bath solution contain no Mg^2+ ^and MK801 (10 μM), CNQX (20 μM) and nimodipine (5 μM) was supplemented.

To verify that the both types of responses reflect activation of the same population of cation channels we designed a protocol to first activate the currents using a relatively long (30-180 sec) application of NM in the presence of control concentrations of divalent cations. This was followed by short period of washout of NM. Figure 4C shows that a brief NM-washout lead to an enhancement of TRPM7 currents that was subsequently suppressed when NM was re-introduced (n = 5), indicating the NM-activated current is blocked by NM. This suggests that NM both blocks TRPM7 currents and paradoxically activates it in the presence of physiological concentrations of divalent cations. If NM-activated currents and TRPM7 currents share the same identity, then Ca^2+ ^per se should affect NM-activated currents. Therefore, we tested if high Ca^2+ ^blocks the NM-activated current. Indeed, addition of 10 mM Ca^2+ ^inhibited NM-activated currents and moreover the presence of 10 mM occluded the activation of NM-activated current (Figure 5A). The amplitude of the NM-activated current was inversely depend upon the concentration of extracellular Ca^2+^: at 10 mM Ca^2+ ^NM induced little current; at 2 mM Ca^2+ ^NM slowly induced a relatively small current; and at 0.1 Ca^2+ ^mM NM rapidly evoked a large current that could be readily blocked upon re-application 10 mM Ca^2+ ^(Figure 5B, n = 6). NM-induced currents were also inhibited by 10 μM Gd^+3 ^(Figure 5C, n = 5) consistent with its identification as TRPM7 currents [6]. These data indicate that NM activates the same currents as the response to low divalent cations solutions.

### NM also induces paradoxical entry of Ca^2+^

We used Ca^2+ ^imaging to test if NM-activated currents lead to Ca^2+ ^influx as do those activated by applications of low concentrations of Ca^2+^. Figure 5D shows at in the presence of 2 mM Ca^2+^, NM induced an increase in the intracellular concentration of Ca^2+^. However, increasing extracellular Ca^2+ ^to 5 mM substantially (p < 0.01) attenuated the NM-induced Ca^2+ ^increase as it also depressed NM-induced inward currents.

### NM and divalent cation block of recombinant TRPM7 channels in HEK293T cells

**Figure 6:**
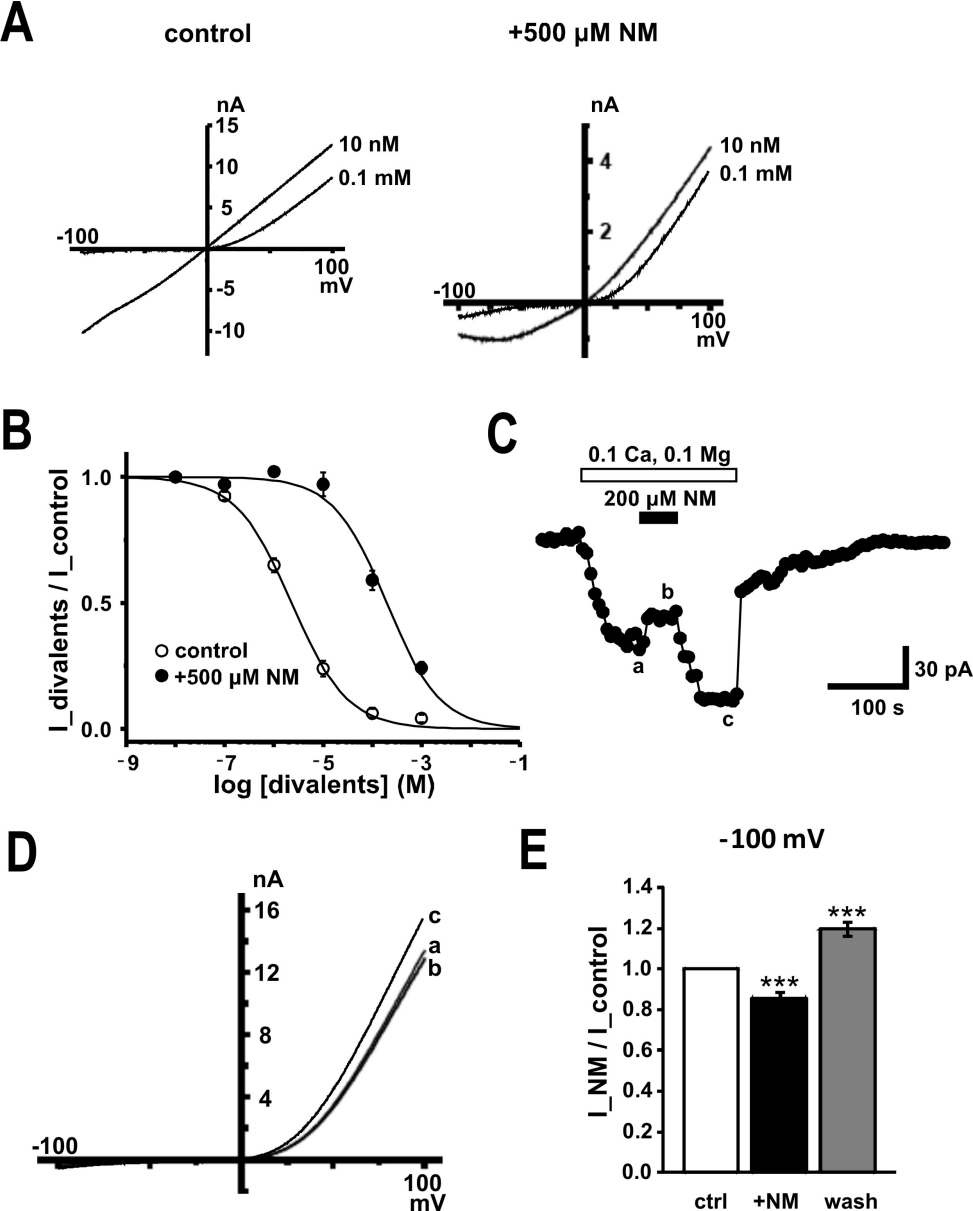
**Effects of NM on the divalent cation sensitivity of TRPM7 currents**. (A) representative *I-V *relationships for currents in response to voltage ramps (50 ms duration) from +100 mV to -100 mV (*V_h _*= 0 mV) recorded with extracellular divalents at 10 nM and 0.1 mM in the absence (left) and presence of 500 μM NM (right). B, dose response curve for the inhibitory effect of divalents on TRPM7 current. Concentration response curve of NM inhibition at +100 mV in the absence of NM; IC_50_: 2.3 ± 0.2 μM; hill coefficient: 0.8; n = 9 and in the presence of NM; IC_50_: 200 ± 40 μM; hill coefficient: 0.8; n = 8. C, NM potentiates TRPM7 channels in TRPM7-transfected HEK293T cells under ramp-clamp (every 5 s at 4 mV/ms). Representative peak inward currents recorded at -100 mV (circle) (V_h _= -60 mV) in the presence of 1 mM Ca^2+ ^and 1 mM Mg^2+^. D, representative *I-V *relationships of currents evoked by 0.1 mM Ca^2+ ^and 0.1 mM Mg^2+ ^in response to voltage ramps before (a), during (b), and after (c) NM application. E, bar graph showing the potentiated ratio by NM on the TRPM7. n = 11, ***, p < 0.01 vs. control.

We next returned to recordings of recombinant TRPM7 channels in HEK293T cells to determine if NM similarly interacted with the divalent cation block of these currents (Figure 6). The effect of NM on the block of TRPM7 currents was examined on the inhibition of these currents by extracellular divalent cations (10 nM versus 0.1 mM) (Figure 6A &6B). The presence of 500 μM NM substantially reduced the blocking potency of this divalent cation (Figure 6B) and is consistent with our observations in cultured hippocampal neurons. Furthermore, an enhancement of TRPM7 currents by NM was also observed (Figure 6C, D &6E).

### NM analogues block TRPM7 currents in cultured hippocampal neurons

**Figure 7:**
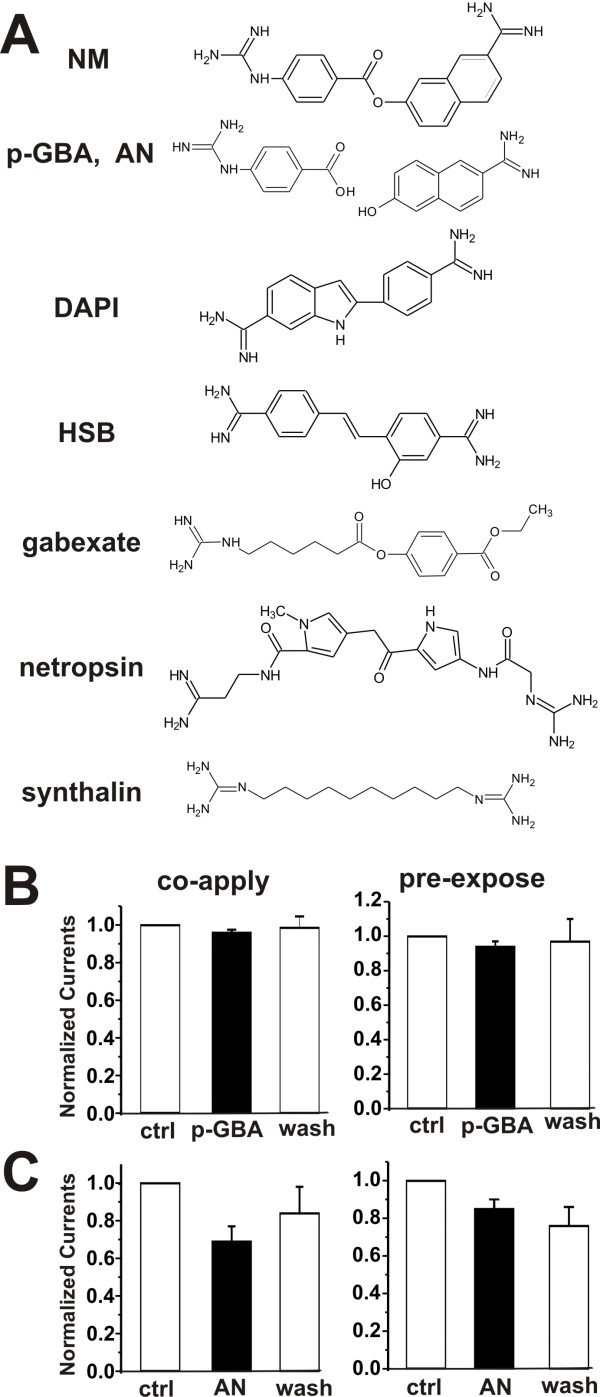
**p-GBA and AN have no or weak effects on TRPM7 current in hippocampal neurons**. A, chemical formulas of NM, p-GBA/AN, DAPI, HSB, gabexate, netropsin, synthalin. B, left, bar-graph of normalized current showing co-applied p-GBA (1 mM) did not block TRPM7 current. I _p-GBA _/I _control _was 94 ± 3%, p = 0.23, n = 4. Right, bar-graph; pre-exposed p-GBA (1 mM) did not potentiate TRPM7 current. I _p-GBA _/I _control _was 96 ± 1%, p = 0.86, n = 3. C, left, co-applied AN (1 mM) slightly blocked TRPM7 current. I _AN _/I _control _was 69 ± 29%, p = 0.03, n = 6. Right, pre-applied AN (200 μM) did not potentiate TRPM7 current. I _AN _/I _control _was 85 ± 5%, p = 0.6, n = 5.

NM is a linear di-cation characterized by an amidine group at one terminus and a guanidine group at the other [20] (Figure 7A). NM is an ester conjugate that can be rapidly hydrolyzed *in vivo *into AN and p-GBA by esterases in the liver and blood. We next tested whether these metabolites mimic NM's effect on TRPM7. p-GBA (up to 1 mM) neither inhibited (through co-application) nor potentiated (pre-exposed application) TRPM7 current (Figure 7B). Co-applied AN (1 mM) only partially inhibited TRPM7 and pre-applied AN produced no potentiation (n = 5, Figure 7C). These results suggest that the both sides of NM are required for the modulation. As NM is a protease inhibitor, we next asked if other linear proteases inhibitors, which share some structural similarity with NM, might modulate TRPM7. We tested protease inhibitors leupeptin and gabexate (Figure 7A) (only having a positive-charged guanidine group at one terminus). Leupeptin (200 μM) had no inhibitory or potentiating effects (not shown). Gabexate (200 μM) only partially inhibited (45 ± 3%, n = 6) TRPM7 current and failed to potentiate the currents (not shown). These results imply that to reach full potency of modulation the di-cationic module of NM appears to be essential. To further examine if the di-cationic structure is sufficient for the modulation we assessed netropsin (Figure 7A), a basic polypeptide isolated from *Streptomyces netropsis*. Netropsin is also a di-cation which, like NM, possesses an amidine group at one end and a guanidine group at the other. We found netropsin (200 μM) neither blocked the current when co-applied, nor potentiated it when pre-exposed (not shown), indicating not all di-cationic molecules affect TRPM7 current and specific structure of di-cations is required. We also found that synthalin (200 μM), a complete linear double-charged bi-guanidine, did not block or potentiate TRPM7 (not shown). Thus, double-charged termini are not sufficient for modulating the TRPM7 and confirm the typical inner motif of NM is important.

**Figure 8:**
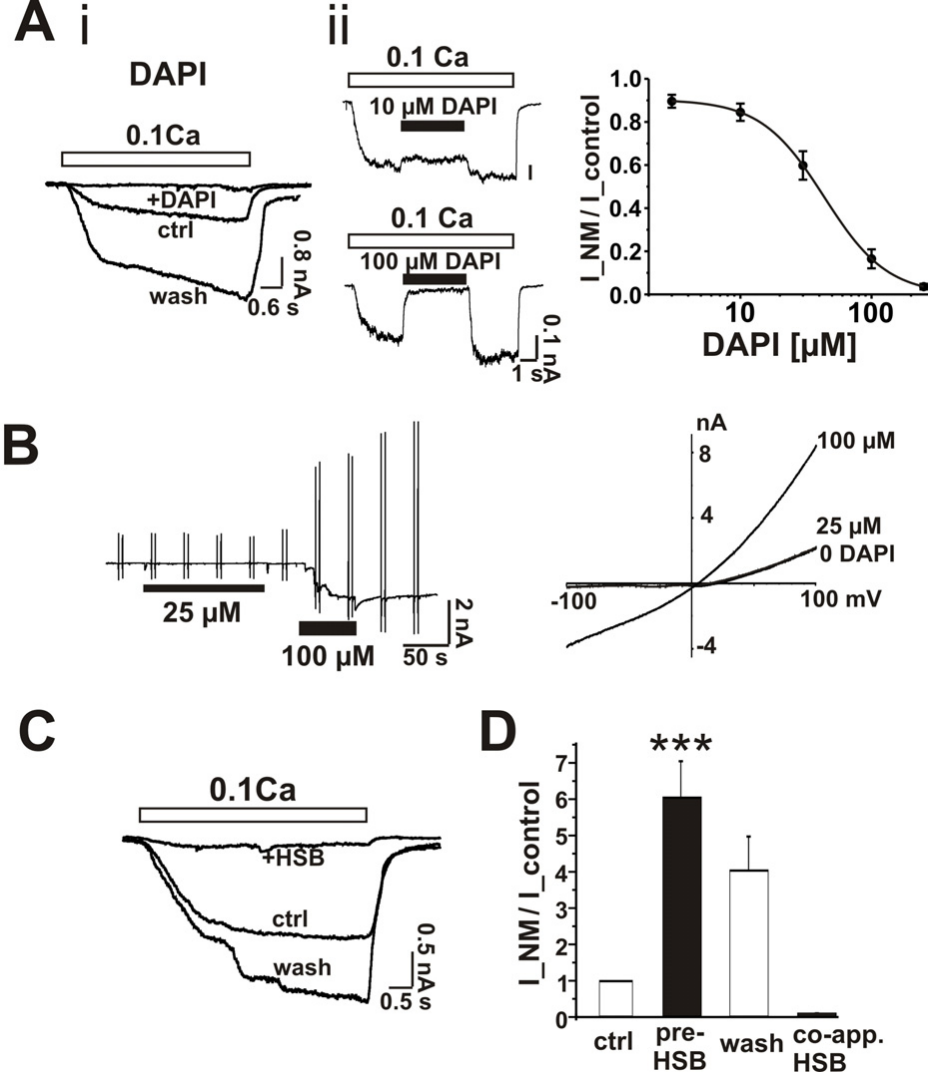
**DAPI and HSB strongly modulate TRPM7 current in hippocampal neurons**. A, i, DAPI (250 μM) completely and reversibly blocked TRPM7 current (evoked by 0.1 mM Ca^2+ ^and 0.1 mM Mg^2+^) in cultured hippocampal neuron, n = 5. ii, left, representative current traces of TRPM7 current blocked with various concentrations of DAPI. DAPI was acutely applied and washed out as indicated by black bar. DAPI rapidly blocked TRPM7 current. iii, concentration response of DAPI blockade of TRPM7 current. IC_50_: 38 ± 8 μM; hill coefficient: 1.8; n = 5. B, DAPI activated TRPM7 at 2 mM Ca^2+ ^and 1 mM Mg^2+^, voltage ramps (-100 mV to 100 mV). Right, *I-V *plot. 100 μM DAPI have strong effect while 25 μM was without effect, n = 5. C, and D, HSB has similar effect as NM on TRPM7. C, co-applied HSB (200 μM) blocks TRPM7 current. D, pre-exposed HSB (200 μM) potentiated TRPM7 current by 6.0 ± 1.0 fold, n = 6, p < 0.01; while co-applied HSB inhibited 90 ± 1% of TRPM7 current, n = 5.

To verify this we tested several compounds with benzene rings on their outer edge such as DAPI and HSB. Both DAPI and HSB are di-cations but with amidine groups at both termini. DAPI completely and reversibly blocked TRPM7 current with an IC_50 _of 38 μM but also directly activated the current in the presence of physiological concentrations of divalent cations (Figure 8A&8B). Co-applications of HSB (200 μM) inhibits TRPM7 current and pre-exposed HSB (200 μM) strongly potentiates (by 6.0 ± 1.0 fold, n = 6) it (Figure 8C). HSB also directly activated the currents in the presence of physiological concentrations of divalent cations (not shown). The effects of DAPI and HSB on TRPM7 were similar to those of NM indicating that more than a di-cationic structure is required for this modulation. We also examined the actions of NM on CA1 neurons in the hippocampal slice in order to confirm whether or not NM was capable of inhibiting and activating this current in CA1 neurons *in situ*. The diffusion barriers presented in the slice preparation preclude very rapid changes in divalent cation concentrations but prolonged solution changes can be used to activate TRPM7 currents in this preparation [19]. Lowering concentrations of divalent cations induced a slowly activating inward current that was attenuated by the co-perfusion of NM (not shown).

## Discussion

Reductions in the concentration of divalent cations dramatically enhance inward currents mediated by recombinant TRPM7 channels, expressed in cell lines as well as in hippocampal neurons [6,21,22]. This enhancement is highly dependent upon membrane potential and reflects, at least in part, the inhibition of TRPM7 monovalent cation flux by extracellular divalent cations. Therefore, current flow through these channels is not only blocked but also carried by divalent cations such as Ca^2+ ^and Mg^2+^.

NM is a commonly used anti-inflammation and anti-coagulation compound [20,23-25]. Accumulating evidence suggests that besides conventional clinical usage NM also shows a myriad of other beneficial effects such as liver protection [26,27], ion channel blockade [28], and pain relief [29]. Our study shows that NM, as well as DAPI and HSB, reversibly block TRPM7 currents in HEK293T cells and TRPM7 currents in cultured hippocampal neurons, provided extracellular divalent cation concentrations are low. In contrast, the NM metabolites, p-GBA and AN were without effect.

We also found that NM inhibited TRPM7 currents expressed in HEK293T cells in direct inverse relationship to the extracellular concentrations of Ca^2+ ^and Mg^2+^. In the absence of extracellular divalent cations, NM caused a potent and voltage-dependent inhibition of TRPM7 currents which mimicked the effects of the divalent cations themselves. The NM-induced inhibition was occluded as the extracellular concentrations of divalents were increased suggesting that NM competes with divalent cations for the block of TRPM7 channels.

In cultured hippocampal neurons we demonstrated that NM, and related compounds, cause a strong membrane potential-dependent inhibition of TRPM7 currents as well as an inhibition the excitatory responses evoked by lowering extracellular concentrations of divalent cations. In previous publications we showed that these currents are at least mediated in large part, if not entirely by TRPM7 channels, as they are negatively correlated with a selective knockdown of TRPM7 protein and messager both in cultured hippocampal and in vivo CA1 pyramidal neurons [5-7]. TRPM7 and TRPM7-like currents are both partially inhibited by 2-APB and neomycin as well as by Ca^2+^, Mg^2+^, Gd^3+ ^and La^3+ ^[6,10,30]. The potency of the block in HEK293T cells and cultured neurons is similar and also supports the substantial involvement of endogenous TRPM7 channels in the responses of neurons to low concentrations of divalent cations [6]. TRPM7 proteins have also been reported to form functional heteromeric channels with TRPM6 and these channels demonstrate somewhat different characteristics than homomeric TRPM7 channels [31]. However, we cannot rule out the possibility that other types of channels (e.g. hemi-gap channels), that are also sensitive to extracellular divalent cations, may also contribute in part to the response of hippocampal pyramidal neurons to brief applications of divalent free solutions [21,22].

Pre-applications of NM prior to reducing extracellular divalents lead to an unanticipated enhancement of the TRPM7 currents. Furthermore, applications of relatively high concentrations of NM were able to directly evoke TRPM7 currents in the presence of intermediate concentrations of divalent cations. Both of these effects were dependent upon membrane potential as well as the extracellular concentrations of divalents. These results demonstrate that NM, and divalent cations likely compete for regulation of TRPM7 currents. Comparing the structure of NM, HSB and DAPI, we note some structural commonalities, for example they are linear dications characterized either by amidino or guanidino groups at both termini together with a variable bulk group (benzene rings) on the outer edge of the molecule. Their ability to mimic the actions of Ca^2+ ^and Mg^2+ ^in the modulation of TRPM7 suggests that they can bind to the same sites on the underlying channel proteins. A parsimonious and consistent interpretation is that NM competes with the binding of divalent cations. At hyperpolarized membrane potentials NM can block these channels when divalent cation concentrations are low but it can also displace divalent binding with the concentrations are higher. This interpretation is consistent with observations that protons also compete with divalent cations in TRPM7 channels to dramatically enhance currents [14]. It is also consistent with our previous observations that protons can inhibit TRPM7 currents in the absence of divalent free cations [17]. At pH 7.4 both the glutamate at 1052 and the aspartate at 1054 are important for the inhibition of divalent cations [15]. NM may be able to access 1052 whereas the deeper 1054 site located in narrowing of the pore is inaccessible [32].

Netropsin, like NM, contains a guanidine group at one end and an amidine group at the other but has a longer structure and is less symmetric than NM. Netropsin had no effect on TRPM7 currents suggesting the length, the relative overall symmetry or the position of bulky group might be important for TRPM7 modulation. The data with synthalin is also consistent with this argument. Both leupeptin and gabexate are protease inhibitors and do not strongly affect TRPM7 showing that protease inhibitors having a guanidine group do not necessarily modulate TRPM7 currents; although, NM and gabexate inhibit a similar spectrum of serine proteases [33].

The capacity of NM, HSB and DAPI to block TRPM7 current suggests a molecular structure which might be explored for the development of agents that block the contributions of these currents to the pathophysiological over activation of central neurons in epilepsy and/or ischemia. Substantial, but transient decreases in extracellular divalent cations are commonly observed in the CNS during pathological events such as epileptic seizures and ischemic conditions [34], and low Ca^2+ ^enhances membrane excitability [10,16]. Compounds based on NM might be anticipated to have little effect until there was a large and pathological decrease in the extracellular concentrations of divalent cations. In this situation they might be anticipated to block TRPM7 currents and therefore reduce the deleterious effects of an influx of Ca^2+ ^and provide a unique mechanism that targets the inappropriate activation of TRPM7 channels. However, the ability of NM to compete with divalents for TRPM7 currents would also potentially predispose activation of TRPM7 currents resulting in enhancement of the currents. In other words they would greatly exacerbate the effects of lowered divalent cations during pathological conditions; and, they would potentially prove excitotoxic rather than neuroprotective. However, in their role as potential activators of TRPM7 they could be used to probe physiological functions these channels in hippocampal neurons as well as their possible role during synaptic transmission at synapses where Ca^2+ ^are likely to fall to very low concentrations [35,36]. Thus, our results provide information about a molecular motif (template) that could be employed to design antagonists or agonists of TRPM7.

## Abbreviations

TRPM: transient receptor potential melastatin; NM: nafamostat mesilate (6-amidino-2-naphthyl-4-guanidinobenzobate dimethanesulfonate); DAPI: Dicationic 4,6-diamidinophenylindole; HSB: hydroxystilbamidine; p-GBA: p-guanidinobenzoic acid; AN: 6-amidino-2-naphthol.

## Competing interests

The authors declare that they have no competing interests.

## Authors' contributions

XC and TN contributed equally to this study. XC carried out the electrophysiological recordings on cultures of hippocampal neurons. ML carried out the calcium imaging. TN performed electrophysiological recordings on TRPM7-expressing HEK cells. XC, TN, YM, BAO, MFJ, ZX, JFM participated in the design and coordination of the study and drafted the manuscript. All authors have read and approved the final manuscript.
